# Assembly of *Francisella novicida* Cpf1 endonuclease in complex with guide RNA and target DNA

**DOI:** 10.1107/S2053230X1700838X

**Published:** 2017-06-20

**Authors:** Pablo Alcón, Guillermo Montoya, Stefano Stella

**Affiliations:** a Protein Structure and Function Programme, The Novo Nordisk Foundation Center for Protein Research, Faculty of Health and Medical Sciences, University of Copenhagen, Blegdamsvej 3b, 2200 Copenhagen, Denmark

**Keywords:** crystallization, protein–DNA interaction, protein–RNA interaction, data collection, genome editing, CRISPR–Cas

## Abstract

Bacteria and archaea use the CRISPR–Cas system as an adaptive response against infection by foreign nucleic acids. Owing to its remarkable flexibility, this mechanism has been harnessed and adopted as a powerful tool for genome editing. The CRISPR–Cas system includes two classes that are subdivided into six types and 19 subtypes according to conservation of the *cas* gene and loci organization. Recently, a new protein with endonuclease activity belonging to class 2 type V has been identified. This endonuclease, termed Cpf1, in complex with a single CRISPR RNA (crRNA) is able to recognize and cleave a target DNA preceded by a 5′-TTN-3′ protospacer-adjacent motif (PAM) complementary to the RNA guide. To obtain structural insight into the inner workings of Cpf1, the crystallization of an active complex containing the full extent of the crRNA and a 31-nucleotide dsDNA target was attempted. The gene encoding Cpf1 from *Francisella novicida* was cloned, overexpressed and purified. The crRNA was transcribed and purified *in vitro*. Finally, the ternary FnCpf1–crRNA–DNA complex was assembled and purified by preparative electrophoresis before crystallization. Crystals belonging to space group *C*222_1_, with unit-cell parameters *a* = 85.2, *b* = 137.6, *c* = 320.5 Å, were obtained and subjected to preliminary diffraction experiments.

## Abstract 2

The purification, reconstitution and crystallization of a complex of *F. novicida* Cpf1 with CRISPR RNA and target DNA is described.

## 1. Introduction   

The prokaryotic adaptive immune system CRISPR–Cas (clustered regularly interspaced short palindromic repeats and CRISPR-associated proteins) provides many bacteria and most archaea with a functional defence mechanism against foreign genetic material such as plasmids and phages (Marraffini, 2015 ▸; Wright *et al.*, 2016 ▸).

Ever since the early characterization of the system, its simplicity and versatility have led to the development of a thriving field that has spread out to essentially every discipline from molecular and cell biology to genetics. Initially described simply as a theoretical concept (Mojica *et al.*, 2005 ▸), the CRISPR–Cas system has experienced an unstoppable rise, unfolding as a boundless tool for genome editing (Jinek *et al.*, 2012 ▸), and its immense versatility can be exploited for multiple applications (Doudna & Charpentier, 2014 ▸). Through RNA-guided recognition, the CRISPR–Cas system can bind and cleave virtually any given target DNA sequence (Marraffini, 2015 ▸). Structural studies have been crucial in revealing the intimate details of the mechanism of interaction between the nuclease and the substrate (Wiedenheft *et al.*, 2009 ▸), enabling structure-guided engineering to improve target specificity and to alter the protospacer-adjacent motif (PAM) requirements (Kleinstiver *et al.*, 2016 ▸; Slaymaker *et al.*, 2016 ▸).

**Figure 1:**
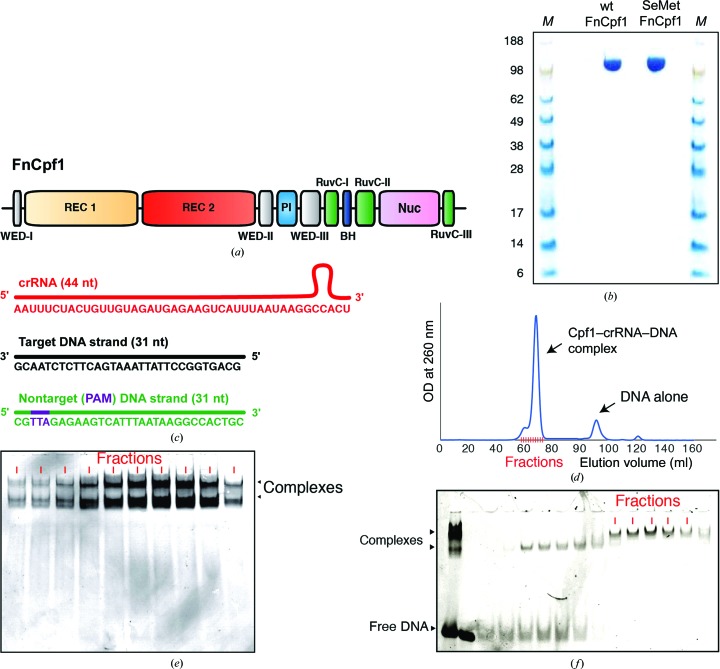
FnCpf1, crRNA and template DNA. (*a*) Domain organization of FnCpf1. (*b*) SDS–PAGE gel showing purified wild-type (wt) FnCpf1 and selenomethionine-derivatized FnCpf1 (SeMet FnCpf1). Lanes *M* contain molecular-weight markers (labelled in kDa). (*c*) Schematic representation of the CRISPR RNA (crRNA) and the target and nontarget (PAM) DNA strands used in assembly of the complex. (*d*) Native PAGE gel showing the fractions corresponding to the Cpf1–crRNA–DNA complex purified by gel-filtration chromatography. (*e*) Separation profile of the ternary Cpf1–RNA–DNA complex by gel-filtration chromatography (see §[Sec sec2]2). (*f*) Native PAGE gel showing the purification of the FnCpf1 complex by preparative vertical-tubular electrophoresis (see §[Sec sec2]2). Native PAGE gel showing the fractions corresponding to the Cpf1–crRNA–DNA complex purified by gel-filtration chromatography; the fractions pooled and used for crystallization are indicated.

According to their molecular architectures, the different members of the CRISPR–Cas system have been classified into two classes: class 1 members encompass several effector proteins, whereas class 2 systems use a single element (Makarova *et al.*, 2015 ▸). Cpf1 (CRISPR from *Prevotella* and *Francisella*) has been described as a new member of the class 2 type V CRISPR–Cas endonucleases that is present in a number of bacterial genomes (Zetsche *et al.*, 2015 ▸) and possesses a range of particular features that have led to its emergence as an encouraging choice for genome-editing applications (Fig. 1 ▸ *a*; Fonfara *et al.*, 2016 ▸). Firstly, Cpf1 uses 42–44 nt RNA and an additional tracer RNA (tracrRNA) is not needed (Zetsche *et al.*, 2015 ▸); this RNA guide is significantly shorter and simpler than the RNA pair [CRISPR RNA (crRNA) and tracrRNA, or a single-guide RNA (sgRNA) in engineered variants] required by Cas9 (Deltcheva *et al.*, 2011 ▸). Moreover, Cpf1 possesses the ability to process its own RNA template, which is cleaved in a sequence-dependent and structure-dependent fashion from the spacer repeat sequence (Yamano *et al.*, 2016 ▸). Secondly, the short and conserved PAM recognized by Cpf1 is a T-rich sequence (Zetsche *et al.*, 2015 ▸) instead of the G-rich motif needed by CRISPR–Cas (Deltcheva *et al.*, 2011 ▸), which might be useful for targeting A/T-rich genomes. The final, and probably the most interesting, unique feature of Cpf1 is the creation of a staggered double-strand break, with a 4 or 5 nt 5′-overhang in its PAM-distal target site (Zetsche *et al.*, 2015 ▸), as opposed to the blunt ends generated by Cas9 within the PAM-proximal target site (Garneau *et al.*, 2010 ▸). For these reasons, Cpf1 is emerging as an upgraded version of the CRISPR–Cas system, which may supplement the growing genome-editing toolbox and open up a wealth of new biotechnological and therapeutic applications.

Recent structural studies have started to shed light on the molecular mechanisms of catalysis by Cpf1. In these studies, the crystal structures of Cpf1 from *Lachnospiraceae bacterium* (LbCpf1; Dong *et al.*, 2016 ▸) in complex with crRNA and from *Acidoaminococcus* sp. (AsCpf1; Yamano *et al.*, 2016 ▸) in complex with crRNA and a truncated portion of target DNA containing the 4 nt PAM sequence have been solved. However, the structures of these complexes offer a snapshot of the target readout and therefore still lack the mechanistic insights necessary to fully describe how the recognition, unzipping and cleavage of the target DNA are achieved. Therefore, we set out to resolve this question by expressing, purifying, reconstituting and assembling FnCpf1 with its crRNA and a 31 bp dsDNA target *in vitro*.

## 2. Materials and methods   

### 2.1. Macromolecule production   

#### 2.1.1. Protein expression and purification   

The gene encoding full-length (residues 1–1300) Cpf1 from *F. novicida* U112 (FnCpf1) was obtained from Addgene (plasmid No. 69975, plasmid name pY003 -pFnCpf1_min). The target locus was amplified by PCR using the specific primers FnCpf1-Forward (CGTATGTTAGGAGGTCTTTCATATGTCAATTTATCAAG) and FnCpf1-Reverse (GATCTGGATCCGTT­ATTCCTATTCTGCACGAACTC) to clone the PCR product into the expression vector pET-21a (catalogue No. 69740-3, EMD Biosciences). The PCR product and the destination vector were subjected to double digestion with the restriction enzymes NdeI and BamHI (New England Biolabs). The digestion products were gel-purified and ligated together using T4 DNA ligase (New England Biolabs) to generate a pET21-FnCpf1 plasmid encoding FnCpf1 fused to a C-terminal hexahistidine tag (His_6_ tag; Table 1 ▸).

*Escherichia coli* BL21 Star (DE3) cells containing the pRare2 plasmid (which supplies seven tRNAs recognizing rare codons) were transformed with the target construct pET21-FnCpf1. A single colony carrying both plasmids was inoculated into 5 ml Luria broth (LB) containing 50 µg ml^−1^ ampicillin and 25 µg ml^−1^ chloramphenicol and incubated overnight at 310 K with shaking at 200 rev min^−1^. 1 ml of the overnight culture was transferred into 1 l fresh LB containing 50 µg ml^−1^ ampicillin and 25 µg ml^−1^ chloramphenicol and incubated at 310 K with shaking at 200 rev min^−1^ until an OD_600_ of 0.8 was reached. The culture was then induced by adding 1 m*M* isopropyl β-d-1-thiogalactopyranoside (IPTG) and incubated at 310 K for 3 h before the cells were collected by centrifugation at 9000*g* for 20 min at 277 K and stored at 193 K until further use. To prepare selenomethionine-substituted protein, cells were grown in SelenoMethionine Medium Complete (Molecular Dimensions) including 40 µg ml^−1^ selenomethionine, 50 µg ml^−1^ ampicillin and 25 µg ml^−1^ chloramphenicol at 310 K with shaking at 200 rev min^−1^. When the culture reached an OD_600_ of 0.8, protein expression was induced with 1 m*M* IPTG and incubation at 310 K for 3 h before the culture was harvested by centrifugation at 9000*g* for 20 min at 277 K.

The cell pellets were defrosted and resuspended in lysis buffer [50 m*M* bicine pH 8.0, 150 m*M* KCl, one tablet of cOmplete Inhibitor Cocktail, EDTA-free (Roche) per 50 ml, 50 U ml^−1^ Benzonase, 1 mg ml^−1^ lysozyme, 0.5 m*M* TCEP]. After cell disruption using a French press, cell debris and insoluble particles were removed by centrifugation at 10 000*g* at 277 K. The supernatant was loaded onto a 5 ml Crude HisTrap column (GE Healthcare) equilibrated in buffer *A* (50 m*M* bicine pH 8.0, 150 m*M* KCl, 0.5 m*M* TCEP). After the sample had been loaded, the column was washed with buffer *A* containing 5 m*M* imidazole to prevent nonspecific binding of contaminants to the resin. Elution was performed by applying a step gradient of 10, 25, 50 and 100% buffer *B* (50 m*M* bicine pH 8.0, 150 m*M* KCl, 0.5 m*M* TCEP, 1 *M* imidazole). Enriched protein fractions corresponding to 25 and 50% buffer *B* were pooled together and applied onto a 5 ml HiTrap Heparin HP column (GE Healthcare) equilibrated with buffer *A*. The protein was eluted with a linear gradient of 0–100% buffer *H* (50 m*M* bicine pH 8.0, 1 *M* KCl, 0.5 m*M* TCEP) in ten column volumes. Protein-rich fractions were collected and concentrated (using 100 kDa MWCO Centriprep Amicon Ultra devices) and subsequently loaded onto a HiLoad 16/60 200 Superdex column (GE Healthcare) equilibrated in buffer *A*. The protein peaks were concentrated (using 100 kDa MWCO Centriprep Amicon Ultra devices), flash-frozen in liquid nitrogen and stored at 193 K. The protein concentration was determined using the theoretical molar extinction coefficient at 280 nm calculated from the amino-acid composition. An overloaded SDS–PAGE stained with SimplyBlue (Invitrogen) displayed a highly pure protein preparation.

#### 2.1.2. RNA transcription   

DNA oligonucleotides corresponding to the reverse-complemented sequence of the target site (67 bases in length) and a short T7 priming sequence (24 bases in length) were purchased from Integrated DNA Technologies (IDT). The oligonucleotides were annealed at a final concentration of 20 µ*M* in annealing buffer consisting of 150 m*M* KCl by heating the mixture to 368 K for 10 min followed by a cool ramp to 277 K over 10 min. This partial DNA duplex was used as a template in a transcription reaction carried out by HiScribe T7 Quick High Yield RNA Synthesis (NEB). The reaction was stopped using 2× stop solution (50 m*M* EDTA, 20 m*M* Tris–HCl pH 8.0, 8 *M* urea) and the RNA was denatured at 368 K for 10 min. The transcription product was purified by preparative electrophoresis with a Bio-Rad Model 491 PrepCell apparatus equipped based on a previously described method (Cunningham *et al.*, 1996 ▸) with some modifications. Briefly, a PrepCell was used with a 37 mm internal diameter gel tube using a 9 cm tall 1× TBE (178 m*M* Tris–borate, 4 m*M* EDTA) 15% (19:1) polyacrylamide/7 *M* urea gel at room temperature. The running buffer 1× TBE and the core gel were prewarmed to 323 K. The gel was run at 14 W constant power for 60 min prior to loading the de­natured sample. The sample was eluted at 1 ml min^−1^ in nuclease-free water. The elution was monitored and fraction­ated. 1× TBE/15% polyacrylamide/7 *M* urea gel was used to identify the fractions containing the correct RNA. The fractions were then pooled together and concentrated using Vivaspin 20 3000 MWCO to an OD_260_ of 30–35.

#### 2.1.3. Complex formation and purification by gel-filtration chromatography and vertical-tubular electrophoresis   

For the formation of the complex, the purified FnCpf1 protein was mixed first with crRNA and incubated for 30 min at 293 K and then with the target DNA duplex (target and nontarget DNA oligonucleotides were purchased from IDT) and incubated for a further 60 min at 368 K (the final protein:RNA:DNA molar ratio was 1:1.3:1.7) in reconstitution buffer consisting of 81 m*M* KCl, 38 m*M* bicine pH 8.0, 5 m*M* MgCl_2_ with a final reaction volume of 960 µl.

The reconstituted complex was purified using a HiLoad 16/60 200 Superdex column (GE Healthcare) equilibrated with buffer *E* consisting of 150 m*M* KCl, 50 m*M* bicine pH 8.0, 0.5 m*M* TCEP. The fractions were loaded onto a native PAGE gel and those containing the complex were pooled and concentrated to 7 mg ml ^−1^.

Aternatively, the assembled Cpf1–crRNA–DNA ternary complex was purified by preparative electrophoresis using a Bio-Rad Model 491 PrepCell apparatus equipped with a 37 mm internal diameter gel tube using a 6 cm tall 8%(*w*/*v*) nondenaturing polyacrylamide gel (19:1 ratio of acrylamide:bis-acrylamide) at 277 K. After a 2 h prerun under constant buffer recirculation, the complex was loaded onto the preparative gel. Electrophoresis was performed at a constant power of 10 W using 0.5× TBE (89 m*M* Tris–borate, 2 m*M* EDTA) as the running buffer and eluting at a flow rate of 1 ml min^−1^ in buffer *E* consisting of 150 m*M* KCl, 50 m*M* bicine pH 8.0, 0.5 m*M* TCEP using an ÄKTAprime system attached to the PrepCell. Highly pure and homogeneous complex was separated from free DNA and high-molecular-weight aggregates and immediately concentrated to 7 mg ml^−1^ with a Vivaspin 20 50000 MWCO centrifugal concentrator for subsequent crystallization experiments.

### 2.2. Size-exclusion chromatography coupled with static laser light scattering (SEC-MALS)   

We verified the homogeneity of the ternary FnCpf1–crRNA–target DNA complex by multi-angle light scattering connected in line with SEC (SEC-MALS). SEC-MALS experiments were performed on a Dionex HPLC system with the UV detector linked to a Wyatt DAWN8+ HELEOS eight-angle light-scattering detector and a Wyatt Optilab T-rEX refractive-index detector. SEC was performed on a Superdex 200 10/300 GL column (GE Healthcare) in 50 m*M* bicine pH 8.0, 150 m*M* KCl, 0.5 m*M* TCEP. 50 µl of the Cpf1 complex was injected at 1 mg ml^−1^ concentration at a 0.5 ml min^−1^ flow rate. The *Astra* software (v.6.1.5) was used to collect data from the ultraviolet, refractive-index and light-scattering detectors and to analyse the data using UV extinction coefficients at 280 nm of 0.951 ml mg^−1^ cm^−1^ (protein) and 10 ml mg^−1^ cm^−1^ (nucleic acid) and refractive-index increments (d*n*/d*c*) of 0.185 ml g^−1^ (protein) and 0.170 ml g^−1^ (nucleic acid).

### 2.3. Crystallization   

Initial crystallization screening was performed at 293 K by the sitting-drop vapour-diffusion method, testing a collection of commercially available crystallization screens (The JCSG+ Suite and The Protein Complex Suite from Qiagen, Crystal Screen HT from Hampton Research and Wizard Cryo 1 & 2 from Rigaku Reagents).

In these experiments, 100 nl drops of the Cpf1 complex at 7 mg ml^−1^ were mixed with the same volume of reservoir solution and set up in 96-well iQ plates (TTP Labtech; 70 µl reservoir), testing three different protein:reservoir volume ratios (1:1, 1.2:1 and 1:1.2) using a Mosquito Crystal robot (TTP Labtech, Melbourn, England). The plates were stored and crystal growth was monitored at 293 K using an automated Rock Imager 1000 imaging system and the *Rock Maker* software package for data management (Formulatrix, Bedford, Massachusetts, USA).

After 5 d of incubation, the extensive initial screening rendered a unique hit from well B2 [0.35 *M* sodium thiocyanate, 20%(*w*/*v*) PEG 3350] of the JCSG-*plus* HT-96 screen (Molecular Dimensions). These initial plate-like crystals formed after 3 d of incubation, only achieving modest dimensions (20 × 20 × 5 µm). The protein content in the crystals was verified using the UV-sensitive camera provided by the imager system. Following initial hit identification, crystal growth was optimized using a Dragonfly screen optimizer (TTP Labtech). Further optimization was carried out by setting up 0.25 µl of complex mixed with 0.25 µl of reservoir solution in a hanging-drop setup on 96-well MRC plates (Molecular Dimensions; 90 µl reservoir), rendering large plate-like crystals of around 200 × 200 × 20 µm (Table 2 ▸). Prior to diffraction experiments, SDS–PAGE and silver-staining analysis of washed and dissolved crystals revealed the presence of all of the components in the ternary FnCpf1–RNA–DNA complex.

### 2.4. Data collection and processing   

Crystals were mounted on CryoLoops (Hampton Research) and soaked into a solution composed of the mother liquor supplemented with 30% methyl-2,4-pentanediol prior to flash-cooling using a CryoStream (Oxford Cryosystems). Initial diffraction experiments and data collection were carried out using an EIGER detector on the X06SA beamline, Swiss Light Source, Villigen, Switzerland. X-ray images were recorded with an EIGER detector using the fine-slicing method (Dauter, 1999 ▸) with 0.2° oscillations at 100 K, a wavelength of 1.0 Å and a crystal-to-detector distance of 386 mm (see Table 1 ▸ for data-collection details and statistics). X-ray diffraction data sets were collected to a resolution of 2.9 Å from native protein crystals. Data processing and scaling were accomplished with *XDS* (Kabsch, 2010 ▸) and *AIMLESS* (Evans & Murshudov, 2013 ▸) as implemented in *autoPROC* (Vonrhein *et al.*, 2011 ▸). Based on the diffraction pattern, these crystals belonged to the orthorhombic space group *C*222_1_, with unit-cell parameters *a* = 85.2, *b* = 137.6, *c* = 320.5 Å, α = β = γ = 90° (Table 3 ▸). To determine the packing of the FnCpf1 ternary complex in the asymmetric unit of the crystal, we calculated the Matthews coefficient (Matthews, 1968 ▸), which yielded a *V*_M_ of 2.35 Å^3^ Da^−1^, corresponding to a solvent content of 48%, with one complex in the asymmetric unit. We attempted to solve the structure of the ternary complex by the molecular-replacement method using LbCpf1 and AsCpf1 without success, suggesting that major conformational changes may occur in the ternary complex. Therefore, selenomethionine-derivatized FnCpf1 was produced to obtain experimental phases using the MAD phasing method.

## 3. Results and discussion   

**Figure 2:**
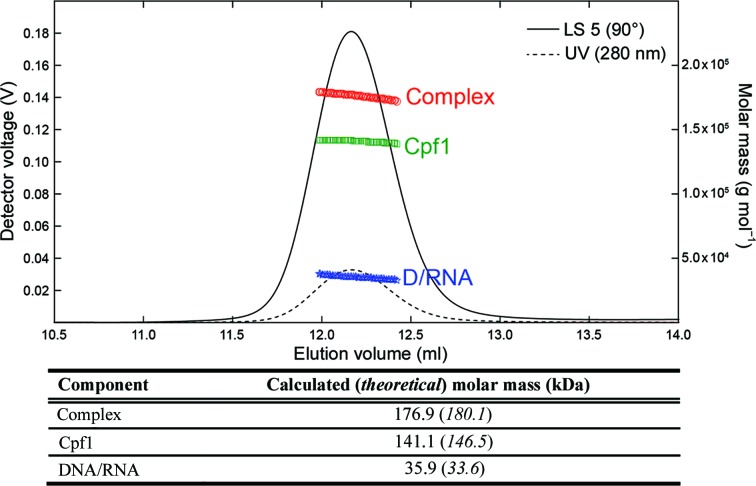
SEC-MALS. The molecular weight of the FnCpf1–crRNA–DNA complex was determined by SEC-MALS-RI-UV. The Rayleigh ratio at 90° (LS 5; continuous line), ultraviolet absorbance (UV; dashed line) and weight-average molar masses (MW) for the complex (red), protein (green) and nucleic acid (blue) are plotted *versus* the elution volume, showing constant molar-mass values over the entire peak width.

**Figure 3:**
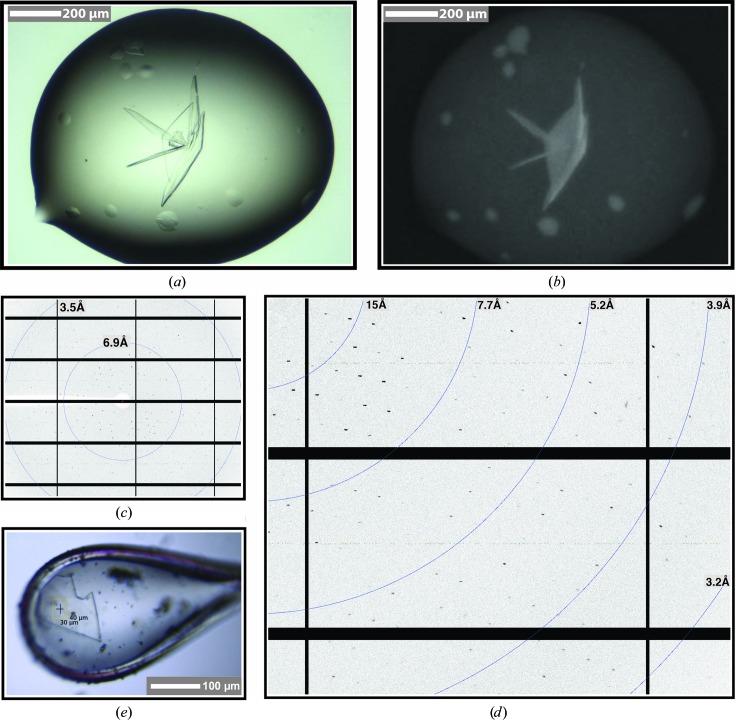
Crystals of the ternary FnCpf1–crRNA–DNA complex. (*a*, *b*) Crystals of the FnCpf1–crRNA–DNA complex obtained in 0.35 *M* sodium thiocyanate, 20%(*w*/*v*) PEG 3350 (see §[Sec sec2]2) under visible (*a*) and UV (*b*) light, revealing the presence of protein. (*c*) Diffraction pattern from crystals of the FnCpf1–crRNA–DNA complex. (*d*) Enlargement of the diffraction image. High-resolution reflections extend to 3 Å. (*e*) An FnCpf1 complex crystal mounted on a CryoLoop for a diffraction experiment.

The previous structures of Cpf1–DNA complexes used a partial double-stranded target; this artificial target stalls the enzyme, which is unable to perform the cleavage reaction because one of the DNA strands is missing (Yamano *et al.*, 2016 ▸). To better understand the catalysis of the Cpf1–crRNA enzyme, we overexpressed and purified FnCpf1 (Fig. 1 ▸ *a*) in both native and selenomethionine-derivatized forms (Fig. 1 ▸ *b*). We used a Bio-Rad Model 491 PrepCell in denaturant conditions to purify the crRNA. We then assembled the complex using the purified components (protein and crRNA) and the target DNA duplex (Fig. 1 ▸ *c*). The traditional purification of the complex by size-exclusion chromatography using a Superdex 200 16/60 (GE Healthcare) produced an apparently homogeneous complex sample (Fig. 1 ▸ *d*); further analysis by native electrophoresis (Fig. 1 ▸ *e*) indicated that the sample contained different species, most likely owing to alternative conformations or catalytic states. Consequently, attempts to crystallize this ternary complex obtained by size-exclusion chromatography were unsuccessful. We then used vertical-tubular native electrophoresis for the first time to purify the FnCpf1–crRNA–DNA target complex with a higher quality (Fig. 1 ▸ *f*). In contrast to classic size-exclusion chromatography, our purification method overcomes the heterogeneity issues described above, providing a highly homogeneous sample (Fig. 1 ▸ *f*). A SEC-MALS experiment showed that this purified complex is homogeneous (Fig. 2 ▸). Furthermore, the superior purity of the Cpf1–crRNA–DNA ternary complex obtained by preparative native electrophoresis was confirmed by its successful crystallization: the complex forms large plate-like crystals of around 200 × 200 × 20 µm in size (Fig. 3 ▸). From the diffraction pattern (Fig. 3 ▸ *d*), these crystals belonged to the orthorhombic space group *C*222_1_, with unit-cell parameters *a* = 85.2, *b* = 137.6, *c* = 320.5 Å, α = β = γ = 90° (Table 3 ▸). Thus, this technique offers the possibility of purifying CRISPR–Cas ternary complexes at specific states of the enzymatic process, providing a powerful tool to investigate the precise molecular events leading to target recognition and catalysis by these RNA-guided endonucleases.

## Figures and Tables

**Table 1:** Macromolecule production

Source organism	*F. novicida* U112
DNA source	Addgene (plasmid No. 69975, plasmid name pY003 -pFnCpf1_min)
Forward primer	CGTATGTTAGGAGGTCTTTCATATGTCAATTTATCAAG
Reverse primer	GATCTGGATCCGTTATTCCTATTCTGCACGAACTC
Expression vector	pET-21a
Expression host	BL21 Star (DE3)

**Table 2:** Crystallization

Method	Sitting-drop vapour diffusion
Plate type	96-well MRC
Temperature (K)	293
Protein concentration (mg ml ^−1^)	7
Buffer composition of protein solution	150 m*M* KCl, 50 m*M* bicine pH 8.0, 0.5 m*M* TCEP
Composition of reservoir solution	0.35 *M* sodium thiocyanate, 20%(*w*/*v*) PEG 3350
Volume and ratio of drop	0.5 µl, 1:1 ratio
Volume of reservoir (µl)	70

**Table 3:** Data collection and processing Values in parentheses are for the outer shell.

Diffraction source	X06SA, SLS
Wavelength (Å)	1.0
Temperature (K)	100
Detector	EIGER 16M X [133 Hz]
Crystal-to-detector distance (mm)	386
Rotation range per image (°)	0.2
Total rotation range (°)	180
Exposure time per image (s)	1
Space group	*C*222_1_
*a*, *b*, *c* (Å)	85.2, 137.6, 320.5
α, β, γ (°)	90
Mosaicity (°)	0.4
Resolution range (Å)	80.12–2.95 (2.96–2.95)
Total No. of reflections	267815 (2811)
No. of unique reflections	39708 (405)
Completeness (%)	98 (98)
Multiplicity	6.7 (7.0)
CC_1/2_	0.99 (0.35)
〈*I*/σ(*I*)〉	7.0 (0.9)
*R*_meas_	0.26 (1.75)
Overall *B* factor from Wilson plot (Å^2^)	65.4
